# Explainable artificial intelligence based analysis for interpreting infant fNIRS data in developmental cognitive neuroscience

**DOI:** 10.1038/s42003-021-02534-y

**Published:** 2021-09-15

**Authors:** Javier Andreu-Perez, Lauren L. Emberson, Mehrin Kiani, Maria Laura Filippetti, Hani Hagras, Silvia Rigato

**Affiliations:** 1 Centre for Computational Intelligence, University of Essex, Colchester, UK; 2 Department of Psychology, Princeton University, Princeton, NJ USA; 3 Centre for Brain Science, Department of Psychology, University of Essex, Colchester, UK

**Keywords:** Neuroscience, Cognitive neuroscience

## Abstract

In the last decades, non-invasive and portable neuroimaging techniques, such as functional near infrared spectroscopy (fNIRS), have allowed researchers to study the mechanisms underlying the functional cognitive development of the human brain, thus furthering the potential of Developmental Cognitive Neuroscience (DCN). However, the traditional paradigms used for the analysis of infant fNIRS data are still quite limited. Here, we introduce a multivariate pattern analysis for fNIRS data, xMVPA, that is powered by eXplainable Artificial Intelligence (XAI). The proposed approach is exemplified in a study that investigates visual and auditory processing in six-month-old infants. xMVPA not only identified patterns of cortical interactions, which confirmed the existent literature; in the form of conceptual linguistic representations, it also provided evidence for brain networks engaged in the processing of visual and auditory stimuli that were previously overlooked by other methods, while demonstrating similar statistical performance.

## Abstract (web summary)

Andreu-Perez et al developed a multivariate pattern analysis for fNIRS data (xMVPA), which is powered by eXplainable Artificial Intelligence (XAI). They demonstrated its application in the context of investigating visual and auditory processing in six-month-old infants and showed that it provided insight into patterns of cortical networks.

## Introduction

Developmental cognitive neuroscience (DCN) is an interdisciplinary field that aims to establish links between the structural changes in the brain, and the corresponding changes in cognitive processes at different time points in development^1^. DCN studies investigate the role of interactions between genetic variations and environmental factors, and their influence on typical and atypical developmental trajectories by examining functional brain development and the increasing specialisation of neural networks^2^.

Although the last two decades have seen remarkable advances in methodologies that allow us to measure functional brain development^1,3^, several challenges still undermine the progress of DCN. The key limitations in developmental research are associated with the limited choice of neuroimaging techniques that can record brain activity non-invasively, and the controversy surrounding the use of standardised and explainable analysis of the data. For example, while magnetic resonance imaging (MRI) may be considered the ad-hoc neuroimaging tool in adult research, it is generally deemed unsuitable for DCN studies since young participants are required to stay still for a substantial amount of time in a confined, restrained environment (but see Blasi et al.^4^ for an example of fMRI developmental study with asleep infants). Similarly, although electroencephalography (EEG) and its associated event-related potential (ERP) methods have been historically employed to examine the psychophysiology of human brain development^5^, the low spatial resolution and the sensitivity to motion artifacts^6^ challenge a comprehensive investigation of the developing brain.

Given the aforementioned methodological limitations, more recently, functional near-infrared spectroscopy (fNIRS) has emerged as a de-facto choice for investigating infant brain development, and its association with cognition and behaviour. fNIRS is a non-invasive, portable, optical neuroimaging method that allows the measurement of cerebral activity using near-infrared (NIR) light with both good temporal (0.01s) and spatial resolution (within 2 cm)^7^. fNIRS has enabled scientists to study asleep and awake infants alike both inside the laboratory settings and in natural environments^8^.

However, despite the recent increased use of fNIRS in DCN, the lack of standard, non-canonical, and explainable frameworks for infant fNIRS data analysis^9^ has limited its potential capacity to map the results to the corresponding spatial activation and contributions between brain regions. A deeper understanding of cortical brain networks for the processing of perceptual stimuli in the developing brain would shed light on the interplay between the physical growth of the activated brain regions and the emergence of new behavioural abilities during brain development^2^. To this end, in this article, we introduce the use of an eXplainable Artificial Intelligence (XAI) inference mechanism for infant fNIRS data that delineates the interaction patterns between brain regions activated in response to perceptual stimuli.

The existent inference frameworks in adult fNIRS analysis involve the use of modelling techniques that assume that signal data coming from all subjects share standard attributes. Typically, these models are based on the assumption that a canonical haemodynamic response function generated in response to a specific stimulus can be represented as a linear combination of several sources (regressors)^10^. Similarly, priors-based modelling, such as seed-based functional connectivity analysis, is heavily dependent on the choice of the channels to be used as a seed^11^. As they stand, the current analysis frameworks are designed for static modelling and therefore cannot be extended to studying brain processes undergoing continuous changes and development. Therefore, as also highlighted in a recent review article^12^, it is necessary to investigate new analytical perspectives in DCN, as models based on adult work are not adequate to study the developing brains. In line with the aim of the present study, Rosenberg and colleagues^12^ encouraged the use of data-driven predictive models to shed light on the neural circuits that give rise to the development of cognition and behaviour.

State-of-the-art machine learning algorithms (e.g., Support Vector Machines (SVM), Random Forest (RF), and neural-network-based approaches) are used for the predictive analysis of neuroimaging data^13^, and are specifically employed to distinguish between classes (stimuli) based on input data (brain responses). However, these paradigms do not explain *what* particular relations of brain activity are prototypical for different stimuli^14–16^. A promising, emerging field for neural data analysis is deep learning^17^. For systems neuroscience, deep learning can provide abstractions of the brain to study neural processing and its anatomical organisation from a theoretical perspective^17^. However, in cognitive neuroscience, and especially with fNIRS, direct statistical models (e.g., linear regression) are the popular choice^18^. This is possibly due to the relatively limited datasets that can be experimentally collected, and the need of cognitive neuroscientists to decode and interpret the complex multivariate patterns of neuroimaging data using straightforward approaches. This limitation is amplified in DCN, where data collection poses additional challenges, such as dealing with infant participants’ compliance with the experiment, and sample sizes are, as a consequence, relatively smaller compared to neuroimaging studies with adults.

In this regard, another analysis paradigm, first introduced for functional MRI data analysis with adults, and recently used to study the infant mind with fNIRS^19^ is multivariate pattern analysis (MVPA)^13^. MVPA deciphers multiple fNIRS channels activity simultaneously to identify informative differences in brain regions’ activation in response to stimuli. By using *N* number of dimensions, arising from *N* number of fNIRS channels, MVPA methods have the potential to identify associations between brain regions, and the corresponding activation levels in terms of distributed patterns, rather than just as measurements of a single source. The two standard classification paradigms used to power MVPA in fNIRS studies are the correlation-based MVPA analysis^19^, and classical machine learning techniques (e.g., LDA^14^, SVM^15^). Although MVPA provides higher sensitivity in comparison to univariate analysis (see Emberson et al.^19^; for an example of MVPA in fMRI data, see Hebart et al.^20^ and Gilbert et al.^21^), the methods used as a basis for MVPA (either correlations or classical machine learning) do not intrinsically outline the prototypical channel regional activation patterns related to each stimulus and their combinations.

In order to overcome these limitations, in the present work, we introduce an XAI learning and inference mechanism for fNIRS MVPA that (1) is not dependent on large datasets, (2) does not rely on a priori model, and (3) provides an explanation for its classification process in the form of patterns of interaction between activated brain regions for the processing of perceptual stimuli. Our eXplainable MVPA (xMVPA) is an XAI inference mechanism for brain haemodynamics data that uses evolutionary learning procedure^22^ to learn the model that drives the MVPA. The functional patterns learnt by the xMVPA, as defined in eq. (1), are captured directly from the input fNIRS measurements. By identifying cortical networks activated for the processing of perceptual information, these patterns can pinpoint the emergence of the specialisation of different brain regions and their interactions, critically contributing to the existent literature of neurodevelopmental trajectories.

A generic nomenclature of a pattern provided by xMVPA for fNIRS data is elucidated in eq. (1):

1 $$\begin{array}{ll}\,{{\mbox{IF}}}\,\ \,{{\mbox{activity}}}\,\ \,{{\mbox{is}}}\,\ CoL\ \,{{\mbox{in}}}\,\ \,{{\mbox{Ch.}}}\,\ X\ &\,{{\mbox{AND}}}\,\ \,{{\mbox{activity}}}\,\ \,{{\mbox{is}}}\,\ CoL\quad \ \,{{\mbox{in}}}\,\ \,{{\mbox{Ch.}}}\,\ Y\ ...\\ &\,{{\mbox{THEN}}}\,\ \,{{\mbox{it}}}\,\ \,{{\mbox{corresponds}}}\,\ \,{{\mbox{to}}}\,\ \,{{\mbox{stimulus}}}\,\ A\end{array}$$

where CoL stands for a conceptual label that denotes the level of activity in a given channel (Ch.), such as *inactive*, *active*, or *very active*.

The inference about the stimulus eliciting the haemodynamic response is made on the basis of the xMVPA patterns defining cortical activation and interactions. A higher classification dexterity of fNIRS data by the explainable patterns of xMVPA can verify that the model has discerned with high accuracy the underlying activation and interactions of the brain regions in response to the presented stimuli. xMVPA automatically performs channel selection for the patterns (i.e., which channels to include in a given pattern) and ensures the generalisation of the inference model by limiting its complexity (i.e., the total number of patterns and their length) as outlined in section xMVPA learning from data.

In the present work, we applied the xMVPA inference mechanism for the explainable classification analysis of infant fNIRS data obtained from an earlier study by Emberson et al.^19^. In this study, fNIRS was used to record 6-month-old infants’ haemodynamic responses to auditory (a toy sound) and visual stimuli (a dynamic red smiley face). The xMVPA obtained six prototypical patterns of brain activation providing new evidence for cortical networks engaged in the processing of visual and auditory stimuli. These patterns give a comparable classification accuracy to the state-of-the-art machine learning algorithms used for MVPA. In addition, xMVPA provides an accessible explanation of its inference, describing the prototypical patterns of functional activation for each stimulus in straightforward terms (if-then rules).

## Results

**Fig. 1:**
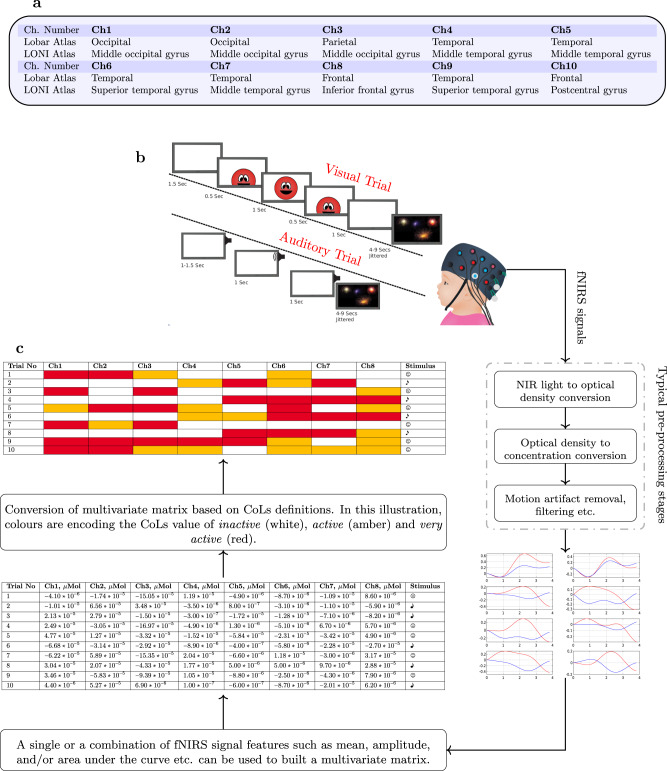
Multivariate pattern matrix construction using fNIRS signals. **a** The average anatomical location of the 10 channels (Chs) after MRI co-registration using Lobar and LONI atlas as delineated in Emberson et al.^19^. **b** A schematic of the Auditory vs. Visual (AV) Experiment in Emberson et al.^19^. The auditory stimulus is a toy sound played for one second, and the visual stimulus is a red smiley face that moves in and out of a box. Please note the location of sources and detectors on the fNIRS cap is hypothetical. **c** A flowchart depicting the steps for the construction of a multivariate pattern matrix with hypothetical numerical neuroimaging data from (arbitrarily chosen) eight fNIRS channels (Chs) associated with two stimulus conditions for ten trials. The numerical multivariate pattern matrix is converted into a conceptual multivariate pattern matrix using conceptual labels (CoLs) of *inactive*, *active*, and *very active*. Please note the numerical values are hypothetical, and since their conversion to the CoLs would depend on which statistical feature of the fNIRS signals has been used, as well as the corresponding definition of the membership function of the associated CoLs (see Fig. 6), no direct inference between the numerical value and the CoL can be made in this illustration.

The xMVPA identifies informative activation patterns by combining the input neuroimaging data from all fNIRS channels of interest into a multivariate matrix. Here, we constructed the multivariate matrix by calculating the mean of the oxygenated haemoglobin (HbO2) signal from each of the 10 channels (see Fig. 1a) in the time-window 4−7 s, following stimulus presentation for each trial (see Fig. 1b). In between the trials, a jittered video of dimmed fireworks was displayed. A grid search was undertaken to find the optimal time window of 4−7 s. In line with previous infant fNIRS studies^23^, and as reported by Emberson et al.^19^, we equally focus on examining the HbO2 signals. Nevertheless, there will be no changes in our proposed xMVPA method for using either or both of the dimensions of the fNIRS signals to construct the multivariate matrix.

Moreover, results of the proposed xMVPA on deoxygenated haemoglobin (deoxy-Hb) signals are provided in subsection 2.1 of the Supplementary File. Deoxy-Hb functional patterns are illustrated in Supplementary Figs. 1 and 2, and a performance comparison with other alternative decoding methods is presented in Supplementary Table 1. In addition, results of the application of xMVPA on an adult fNIRS dataset on mental arithmetic are provided in subsection 2.2 of the Supplementary File. The decoding accuracy is contrasted with the results reported in the dataset’s article^24^ in Supplementary Table 2 and resultant patterns are elucidated in Supplementary Table 3 and Fig. 3.

The ethics approval for the experiment is provided by the Institutional Review Board of the University of Rochester, and informed consent is obtained from the caregivers of the infants^19^. More details on the experiment reproducibility and statistics are provided in section “Statistics and reproducibility”. The reader is referred to the earlier study by Emberson et al.^19^ for more details on the experimental setup, data collection, sample, control, exclusion, and the subsequent pre-processing steps.

The numerical neuroimaging data in the multivariate matrix is then translated into CoLs of brain activation defined as *inactive*, *active*, and *very active* to represent the average activity level of each channel for the time-window considered. A flow chart outlining the steps for generating a multivariate pattern matrix with CoLs is presented in Fig. 1c. The data instances in the multivariate matrix characterised by the CoLs for each trial are subsequently used to train the xMVPA for explainable classification results of the infant data in response to the visual and auditory stimuli. More details of the proposed xMVPA inference mechanism are provided in the section “Methods”: a MVPA method via XAI (xMVPA).

**Fig. 2:**
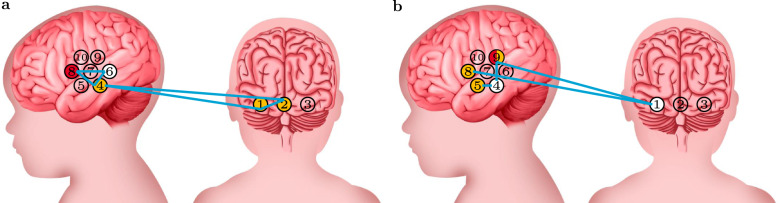
Patterns of cortical networks delineated by xMVPA. The patterns (cyan) identified by the xMVPA delineate the contributions between brain regions evoked by **a** visual and **b** auditory stimuli. The colour of the channels denotes their level of activity: inactive (white), active (amber), and very active (red), and uncoloured for channels that do not belong to any pattern.

xMVPA revealed six functional patterns of interactions between cortical regions using the publicly available DCN dataset of auditory versus visual stimulus processing^25^. The patterns form the inference model for xMVPA as they predict the stimulus (or class) for the brain activity instances (or data instances). These explainable patterns are listed below:

The six patterns provided by xMVPA that outline the brain regions’ activation and interaction for processing visual and auditory information are given below:

$$\begin{array}{ll}{{{{{{{\rm{Pattern}}}}}}}}\ {{{{{{{{\rm{P}}}}}}}}}_{1}:&{{{{{{{\rm{IF}}}}}}}}\ {{{{{{{\rm{Ch1}}}}}}}}\ {{{{{{{\rm{is}}}}}}}}\ Active\ {{{{{{{\rm{AND}}}}}}}}\ {{{{{{{\rm{Ch2}}}}}}}}\ {{{{{{{\rm{is}}}}}}}}\ Active\ {{{{{{{\rm{AND}}}}}}}}\ {{{{{{{\rm{Ch4}}}}}}}}\ {{{{{{{\rm{is}}}}}}}}\ Active\\ &{{{{{{{\rm{THEN}}}}}}}}\ {{{{{{{\rm{stimulus}}}}}}}}\ {{{{{{{\rm{is}}}}}}}}\ Visual\ {{{{{{{\rm{with}}}}}}}}\ {{{{{{{\rm{dominance}}}}}}}}\ {{{{{{{\rm{score}}}}}}}}\ 0.581\\ {{{{{{{\rm{Pattern}}}}}}}}\ {{{{{{{{\rm{P}}}}}}}}}_{2}:&{{{{{{{\rm{IF}}}}}}}}\ {{{{{{{\rm{Ch4}}}}}}}}\ {{{{{{{\rm{is}}}}}}}}\ Active\ {{{{{{{\rm{AND}}}}}}}}\ {{{{{{{\rm{Ch6}}}}}}}}\ {{{{{{{\rm{is}}}}}}}}\ Inactive\ {{{{{{{\rm{AND}}}}}}}}\ {{{{{{{\rm{Ch8}}}}}}}}\ {{{{{{{\rm{is}}}}}}}}\ Very\ Active\ \\ &{{{{{{{\rm{THEN}}}}}}}}\ {{{{{{{\rm{stimulus}}}}}}}}\ {{{{{{{\rm{is}}}}}}}}\ Visual\ {{{{{{{\rm{with}}}}}}}}\ {{{{{{{\rm{dominance}}}}}}}}\ {{{{{{{\rm{score}}}}}}}}\ 0.019\\ {{{{{{{\rm{Pattern}}}}}}}}\ {{{{{{{{\rm{P}}}}}}}}}_{3}:&{{{{{{{\rm{IF}}}}}}}}\ {{{{{{{\rm{Ch1}}}}}}}}\ {{{{{{{\rm{is}}}}}}}}\ Inactive\ {{{{{{{\rm{AND}}}}}}}}\ {{{{{{{\rm{Ch8}}}}}}}}\ {{{{{{{\rm{is}}}}}}}}\ Active\ \\ &{{{{{{{\rm{THEN}}}}}}}}\ {{{{{{{\rm{stimulus}}}}}}}}\ {{{{{{{\rm{is}}}}}}}}\ Auditory\ {{{{{{{\rm{with}}}}}}}}\ {{{{{{{\rm{dominance}}}}}}}}\ {{{{{{{\rm{score}}}}}}}}\ 0.434\\ {{{{{{{\rm{Pattern}}}}}}}}\ {{{{{{{{\rm{P}}}}}}}}}_{4}:&{{{{{{{\rm{IF}}}}}}}}\ {{{{{{{\rm{Ch4}}}}}}}}\ {{{{{{{\rm{is}}}}}}}}\ Inactive\ {{{{{{{\rm{AND}}}}}}}}\ {{{{{{{\rm{Ch5}}}}}}}}\ {{{{{{{\rm{is}}}}}}}}\ Active\ \\ &{{{{{{{\rm{THEN}}}}}}}}\ {{{{{{{\rm{stimulus}}}}}}}}\ {{{{{{{\rm{is}}}}}}}}\ Auditory\ {{{{{{{\rm{with}}}}}}}}\ {{{{{{{\rm{dominance}}}}}}}}\ {{{{{{{\rm{score}}}}}}}}\ 0.406\\ {{{{{{{\rm{Pattern}}}}}}}}\ {{{{{{{{\rm{P}}}}}}}}}_{5}:&{{{{{{{\rm{IF}}}}}}}}\ {{{{{{{\rm{Ch4}}}}}}}}\ {{{{{{{\rm{is}}}}}}}}\ Inactive\ {{{{{{{\rm{AND}}}}}}}}\ {{{{{{{\rm{Ch9}}}}}}}}\ {{{{{{{\rm{is}}}}}}}}\ Very\ Active\ \\ &{{{{{{{\rm{THEN}}}}}}}}\ {{{{{{{\rm{stimulus}}}}}}}}\ {{{{{{{\rm{is}}}}}}}}\ Auditory\ {{{{{{{\rm{with}}}}}}}}\ {{{{{{{\rm{dominance}}}}}}}}\ {{{{{{{\rm{score}}}}}}}}\ 0.239\\ {{{{{{{\rm{Pattern}}}}}}}}\ {{{{{{{{\rm{P}}}}}}}}}_{6}:&{{{{{{{\rm{IF}}}}}}}}\ {{{{{{{\rm{Ch1}}}}}}}}\ {{{{{{{\rm{is}}}}}}}}\ Inactive\ {{{{{{{\rm{AND}}}}}}}}\ {{{{{{{\rm{Ch9}}}}}}}}\ {{{{{{{\rm{is}}}}}}}}\ Active\ \\ &{{{{{{{\rm{THEN}}}}}}}}\ {{{{{{{\rm{stimulus}}}}}}}}\ {{{{{{{\rm{is}}}}}}}}\ Auditory\ {{{{{{{\rm{with}}}}}}}}\ {{{{{{{\rm{dominance}}}}}}}}\ {{{{{{{\rm{score}}}}}}}}\ 0.082\end{array}$$

where dominance score (DS) is in the range (0,1) DS of a pattern indicates the overall information prowess of a given pattern with DS = 1 being the most informative pattern and DS = 0 being the least informative pattern. More details on the metric DS are provided in the section “Methods”: A MVPA method via XAI (xMVPA):

Patterns P_1_ and P_2_ identified interactions between regions involved in the processing of the visual stimulus, as shown in Fig. 2a. Firstly, P_1_ showed a prominent involvement of the occipital cortex, where channel 1 and channel 2 are both classified as *active*. Secondly, both P_1_ and P_2_ identified an *active* status of channel 4, located in the temporal cortex (see Fig. 1a). Finally, P_2_ identified an *inactive* status of channel 6 in the temporal cortex in combination with an *active* status of channel 4 and a *very active* status of channel 8, located in the frontal cortex.

The patterns of interactions in response to the auditory stimulus are shown in Fig. 2b. Here, channels that are active correspond to the prefrontal cortex (channel 8 in P_3_) and temporal cortex (channel 5 in P_4_ and channel 9 in P_5_ and P_6_). The occipital cortex is not engaged in the processing of auditory stimulus as indicated by the *inactive* status of channel 1 in combination with both prefrontal cortex (channel 8 *active* in P_3_) and temporal cortex (channel 9 *active*) in P_6_ activation.

Taken altogether, the patterns identified by the xMVPA show activation over the occipital and temporal cortices for visual stimulus processing and over the temporal cortex for auditory stimulus processing. The patterns also identified activity over the frontal cortex for the processing of both auditory and visual stimuli.

### Results comparison with MVPA

Another important observation from the patterns in P_1_−P_6_ is that no individual channel in the temporal cortex with sufficient decoding accuracy stood out for processing the auditory stimulus presented to the infants in the study, i.e., no channel had stimulus-specific activation (for example, *active* for auditory processing, and *inactive* for visual processing) as reported in Table 1. This is consistent with the correlation MVPA analysis reported in Emberson et al.^19^ (see Table 1). Nevertheless, the MVPA method was unable to specify neither the semantics of such activation difference nor the combination of channels yielding higher decoding, just the independent decoding strength for each channel. The absence of decoding strength in the temporal cortex in response to auditory stimuli might be due to a more diffuse cortical activity^26^, in line with what is suggested by fMRI and fNIRS studies that report widespread activation in response to auditory stimuli, such as sounds^4,27^, in the infant’s brain.

**Table 1:** A comparison of the (Ch) significance and decoding strength found using correlation-based MVPA Emberson et al.^19^ (second row) with the channel activations using conceptual labels (CoLs) of *inactive*, *active*, and *very active* provided by the proposed xMVPA (third row) for the Auditory-Visual (AV) experiment in the study by Emberson et al.^19^.

Anatomical Location	Occipital cortex			Temporal cortex					Pre-frontal cortex	
Activation Level	Ch1	Ch2	Ch3	Ch4	Ch5	Ch6	Ch7	Ch9	Ch8	Ch10
**Significant activation**	✓						✓	✓		✓
**Visual processing**	Active	Active		Active		Inactive			Very active	
**Audio processing**	Inactive			Inactive	Active			Active or very active	Active	

### Decoding performance comparison with black-box models

**Fig. 3:**
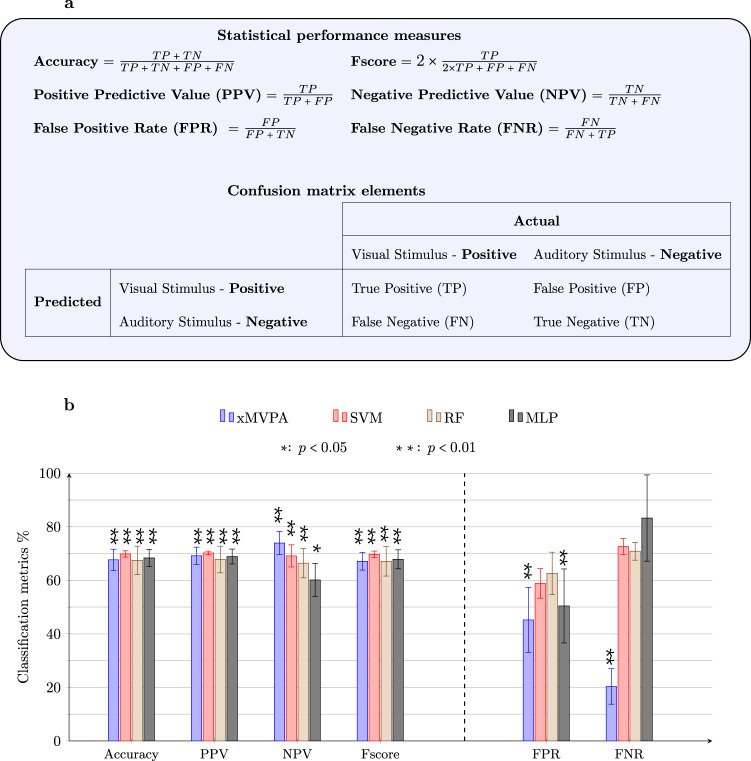
Comparison of statistical performance measures of xMVPA with black-box models. **a** Definition of the statistical performance measures used for comparison of frameworks prowess in driving MVPA. A confusion matrix of binary classification problem, i.e., predicting one of the two stimuli (Visual or Auditory) that best matches the brain activity instances. The visual stimulus is referred to as the positive stimulus and the auditory stimulus as the negative stimulus. **b** A bar chart for the comparison of frameworks driving MVPA including the proposed model xMVPA, Support Vector Machines (SVM), Random Forest (RF), and Multi-Layer Perceptron (MLP) on infant’s unisensory fNIRS dataset. PPV: predictive positive value; NPV: negative predictive value; FPR: false positive rate; FNR: false negative rate with error bars representing the standard deviation. The statistical performance measures that attain statistical significance over the decoding results of a baseline classifier with 5,000 randomly permuted stimulus labels are denoted with asterisks (*). A breakdown of the values and statistics in this figure is detailed in Supplementary Table 4.

A range of statistical performance measures derived from the confusion matrix, outlined in Fig. 3a, are calculated to quantify the performance of the xMVPA patterns. The confusion matrix helps assess the robustness of a given model’s inference mechanism by indicating whether or not the model is ‘confusing’ the classes, i.e., decoding visual stimulus when it is an auditory stimulus (or vice versa). Please note, in Fig. 3a, the visual stimulus is referred to as a positive class, and the auditory stimulus is referred to as a negative class.

The bar graph in Fig. 3b shows a comparison of the statistical performance measures (accuracy, positive predictive value (PPV), negative predictive value (NPV), Fscore, false positive rate (FPR), and false negative rate (FNR) defined in Fig. 3a) between the xMVPA and the state-of-the-art machine learning algorithms SVM, RF, and MLP. The statistical performance measures of accuracy, PPV, NPV, and Fscore for xMVPA are comparable to those obtained for SVM, RF, and MLP. However, the xMVPA outperforms all the other models for the metrics FPR and FNR. The lowest values of FPR and FNR for xMVPA indicate the most robust classification method (also named *decoding model* in MVPA^19^) for the input fNIRS data, i.e., the xMVPA obtains the least fNIRS instances predicted as auditory when they are in factual evoked by visual stimuli and vice versa. Altogether, this comparison confirms that the xMVPA’s patterns clearly discern the differences in the fNIRS instances for the six-month-old brain in response to visual and auditory stimuli.

## Discussion

In the present study, we provide a novel, explainable method for analysing and interpreting infant fNIRS data. The proposed xMVPA is an MVPA based on XAI that provides functional patterns characterised by conceptual labels delineating contributions between brain regions for information processing. We applied the xMVPA to the analysis of a group of 6-month-old infants’ brain activity in response to visual and auditory stimuli^19^, and identified six patterns of cortical networks. Our results showed that the classification accuracy obtained on the infant fNIRS dataset by the proposed xMVPA is comparable to the state-of-the-art machine learning algorithms frequently used for MVPA (e.g., SVM, RF, and MLP; see Fig. 3b), thus demonstrating the validity of our model. This is of critical importance for advancement in DCN because, in contrast to our xMVPA, the classification process of these standard machine learning algorithms is opaque^14,15^ and thus cannot inform our understanding of the developing brain.

The validity and efficacy of our model are also demonstrated against the correlation-based MVPA presented in the previous study by Emberson et al.^19^. As reported in Table 1, channel 1 is the only channel to have both decoding strength in the correlation-based MVPA reported by^19^, and stimulus-specific activation for visual and auditory processing in our xMVPA analysis (see Table 1), i.e., channel 1 is specifically *active* in response to the visual stimulus, but *inactive* in response to the auditory stimulus. This specific pattern of activation is also consistent with the localisation of channel 1 in the occipital cortex, responsible for the processing of visual information^28^. In addition, our xMVPA patterns also delineate the interconnection of channel 1 with other channels (channel 2 and channel 4 in P_1_), uncovering a network of cortical regions for visual processing.

Our xMVPA has identified two brain activity patterns (P_1_ and P_2_) in response to the dynamic visual stimulus presented to the 6-months-old infants in the study. Specifically, we found activation of the occipital cortex and the prefrontal cortex, with partial activation of the temporal cortex.

The activation of the occipital cortex for processing visual information in infancy is well-established in the literature. For example, Wilcox and colleagues^29^ reported activity over the occipital cortex when 6.5-month-old infants were presented with an occlusion event involving objects. Watanabe et al.^30^ showed that 3-month-old infants’ occipital cortex was activated for both dynamic (moving mobile objects) and static visual stimuli (black-and-white checkerboard pattern). Similar to our findings, they also reported activation over temporal and prefrontal cortices in response to the dynamic stimulus. Hence, the patterns P_1_ and P_2_ provided by the xMVPA are in line with the existent literature, suggesting that a specific cortical network of regions involving the occipital, temporal, and prefrontal cortices is involved in the processing of dynamic visual information.

**Fig. 4:**
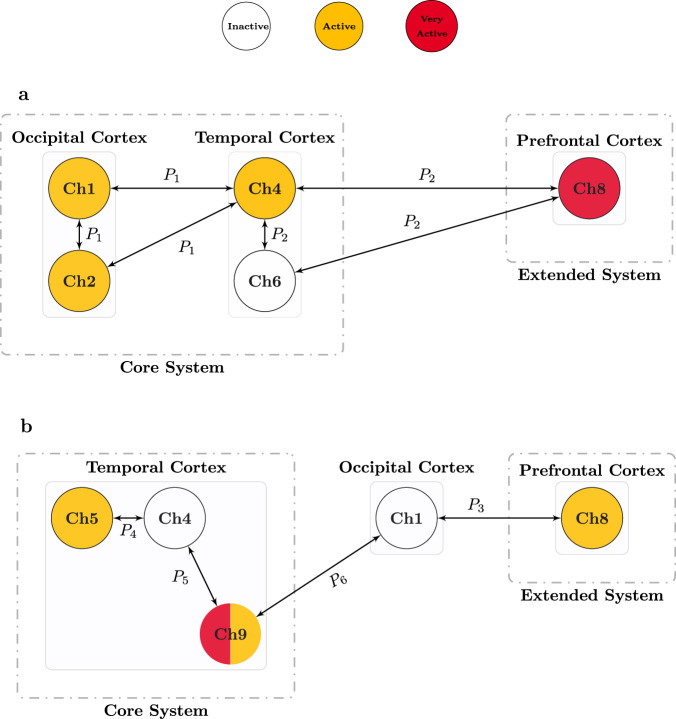
Illustration of cortical networks proposed by xMVPA. **a** A model for face processing in 6-month-old infants, based on the patterns P_1_ and P_2_ revealed by the xMVPA inference mechanism. The proposed model consists of an occipital-temporal network as a core system that undertakes the primary processing of facial features (two eyes above a nose above a mouth), and of the prefrontal cortex as an extended system that processes the emotion associated with the visual stimulus (i.e., associate happiness with a smiley face). **b** A model for non-speech auditory processing in 6-month-old infants, based on the patterns P_3_ to P_6_ revealed by the xMVPA inference mechanism. The proposed model consists of the temporal cortex as a core system for processing non-speech auditory stimuli, and of the prefrontal cortex as an extended system that processes the emotion associated with the auditory stimulus. The colour of the channel’s (Ch) circle is based on its activity level: inactive (white), active (amber), and very active (red).

It is important to note that the dynamic visual stimulus used by Emberson et al.^19^ displayed human facial attributes. Extending previous findings of studies that investigated face processing in young infants^31,32^, we found a specific inter-regional interaction between the occipital and temporal cortices (P_1_) in response to the face stimulus. A similar network of occipital and temporal regions for visual processing is also found in the adult literature^33^. In particular, the occipitotemporal region is identified as a ‘core system’ in the model of the distributed human neural system for face perception in adults^34^. Thus the interaction between occipital and temporal cortices identified in the pattern P_1_ in our study provides evidence for the existence of an equivalent ‘core system’ for face processing in six-month-old infants (Fig. 4a).

In addition, pattern P_2_ identified inter-regional interaction between the prefrontal and temporal cortices. This indicates that infants as young as six months of age recruit an extended neural system for processing social stimuli, such as faces, adding to the existing literature that found similar activations in older infants^35,36^. This is also in line with the ‘extended system’ in the model of face perception in adults^34^, which is a dedicated network over the temporal and prefrontal cortices for processing basic facial emotions.

In line with Emberson et al.^37^, we found no pattern in P_1_ or P_2_ suggesting direct inter-regional interactions between the occipital (channel 1 and channel 2) and the prefrontal cortex (channel 8, inferior frontal gyrus) in response to the visual stimulus. However, previous studies have demonstrated the involvement of the prefrontal cortex during the presentation of visual stimuli in newborns^28^ and 3-month-old infants^30^. While there is evidence supporting the functional role of the prefrontal cortex in the early postnatal period^38^, it is possible that the functional connections between the visual and frontal cortex undergo experience-dependent synaptic pruning during this time^39^ leading to potential functional specialisation in the occipital cortex by 6 months of age^40^. In support to this hypothesis, a study by Homae et al.^41^ demonstrated a decrease in connectivity between prefrontal and occipital cortices from birth to six months. Taken together, the results reported by Homae et al.^41^ and Emberson et al.^37^, as well as the absence of interaction between occipital and prefrontal cortex in the present work, suggest that the role of the prefrontal cortex is not representative in the core processing of visual information at 6 months of age. However, the direct connections with the temporal cortex suggest that the prefrontal cortex may play a role in the extended system for deriving meaning from the visual stimulus. This is in line with the established role of the prefrontal cortex as an overall control unit that receives input from perceptual cortices and generates meaning from the received input^2,42^.

Based on the above discussion on the patterns provided by the xMVPA, a model for the cortical pathways for the processing of visual stimulus in six-month-old infants is presented in Fig. 4a. The model for the developing brain has similar modules and interconnections as the adult neural system for face perception presented by Haxby et al. in^34^ suggesting that by 6 months of age, the cortical activity associated with face processing is already similar to that of mature brains.

A total of four patterns, P_3_ to P_6_, were identified by the xMVPA for the processing of the auditory stimulus. Specifically, while patterns P_4_, P_5_, and P_6_ delineated the involvement of the temporal cortex, the activation of the prefrontal cortex is observed in pattern P_3_. This evidence alings with the literature, whereby non-speech auditory stimuli elicit consistent responses in the infant temporal^43^ and prefrontal cortex^37^.

While activation of the prefrontal and temporal cortices were found, none of the patterns revealed an interaction between these areas. Previous studies with infants reported non-synchronised activity in temporal and prefrontal cortices in response to non-speech auditory stimuli^44,45^, whereas activation in both temporal and prefrontal cortices has been reported in response to speech-like sounds^46,47^. Considering that in the present work, the auditory cue presented to infants was a non-speech stimulus, our results are in line with the literature and suggest that inter-regional interactions between the temporal and prefrontal cortex might be specific to speech-like sounds^46,47^. While this interpretation would fit both with our results and with the available evidence from previous infant research, further studies should use the xMVPA model to test this hypothesis directly.

None of the patterns identified activation of the occipital cortex in response to the auditory stimulus; indeed channel 1 was found *inactive* in patterns P_3_ and P_6_. While this is not surprising, as the occipital cortex is usually recruited in response to visual, rather than auditory stimuli^28^, it is important to point out that this finding further strengthens the validity of our xMVPA model.

Our model also shows a particular activation pattern over the temporal cortex specific to visual vs auditory stimuli. Specifically, the channels of the temporal cortex, which are active in response to the visual stimulus are instead inactive in response to the auditory stimulus, i.e., channel 4 is *active* in P_1_ and P_2_ for visual processing and *inactive* in P_4_ and P_5_ for auditory processing. This confirms the multifaceted role of the temporal cortex in the processing of sensory stimuli thereby, some areas are dedicated to visual processing^29–31,34^ whilst others are associated with auditory processing^4,46,47^.

Based on this body of evidence, with this work, we hypothesise a non-synchronised model for the cortical pathways engaged in the processing of non-speech auditory stimuli in six-month-old infants. This proposed model is composed of a ‘Core’ and an ‘Extended’ system, as shown in Fig. 4b. The temporal cortex will form the core system for processing non-speech auditory stimuli, while the prefrontal cortex will form the extended system for processing the emotion associated with the auditory stimulus. When inactive, the occipital cortex enables the occurrence of these patterns.

Taken together, the patterns P_1_ to P_6_ obtained by the proposed xMVPA have provided not only corroborative evidence for the existent literature for the processing of perceptual information in infants but also revealed new brain regions activation and interactions not yet established for the developing brain. Learning new cortical pathways directly from the neuroimaging data is of fundamental importance in DCN research to shed light on functional brain development in the absence of established assumptions. In this work, we introduced an AI-powered explainable approach to interpreting infant neuroimaging data. The xMVPA, a MVPA for fNIRS data analysis powered by XAI, overcomes important methodological limitations currently present in DCN and represents a stepping stone for furthering our understanding of the functional development of the human brain. The proposed xMVPA is here applied on fNIRS data obtained in response to visual and auditory stimuli in a group of 6-month-old infants^19^. The xMVPA identified six patterns describing cortical activations and inter-regional interactions specific to each of the perceptual stimuli. These patterns corroborated the existing evidence in the DCN literature, while providing further insight into auditory processing in infants.

Given its capability and reliability of identifying patterns of inter-regional interactions for information processing, the xMVPA provides a technical framework for the *Interactive Specialisation* (IS) account proposed by Johnson^2^ for explaining functional brain development. The IS account suggests that postnatal brain development emerges due to the optimisation of interactions between different regions of the brain. In more detail, it suggests that cortical regions interact and compete with each other to acquire their role in new computational abilities, therefore becoming more specialised with development. Critically, the onset of new behavioural abilities is associated with changes in activity over cortical networks and not by the onset of activity in single regions. Future prospective use for xMVPA can be dedicated to identifying changes in the activation of cortical regions and networks that may characterise, directly from the neural data, typical and atypical development. Even more, the employment of the xMVPA to a longitudinal dataset is a promising avenue for the study of developmental brain trajectories in terms of maturation and inter-regional functional interactions^2^. A limitation of the present study is that the cross-sectional nature of the dataset could only inform us on the brain regions involvement for 6-month-old infants.

Future developments of the xMVPA could focus on the inclusion of time information in the multivariate matrix since using a single value such as the mean of the fNIRS signal to construct the multivariate matrix does not retain the time dimension of the fNIRS signal. The proposed xMVPA could therefore be extended to provide complementary time-stamps patterns and map brain regions activation and interactions to a corresponding time after stimulus presentation. This will further enhance the potential of the xMVPA to contribute to the field of DCN critically.

## Methods: a MVPA method via XAI (xMVPA)

The patterns obtained from xMVPA are formed of two parts: the antecedent part, A, and the consequent part, Y, as outlined in eq. (2).

2 $$\,{{\mbox{Pattern}}}\,\ :\ \,{{\mbox{IF}}}\,\ Antecedents\ \,{{\mbox{THEN}}}\,\ Consequent$$

The patterns of activation between fNIRS channels that map interactions among brain regions (antecedents (A)) to particular stimuli (consequent (Y)), are defined as follows in eq. (3):

3 $$\begin{array}{ll}&\,{{\mbox{Pattern}}}\,\ {P}_{q}:\,{{\mbox{IF}}}\,\ N{V}^{1}\ \,{{\mbox{is}}}\,\ Co{L}^{1}\ \,{{\mbox{AND}}}\,\ ...\ \,{{\mbox{AND}}}\,\ N{V}^{n}\ \,{{\mbox{is}}}\,\ Co{L}^{n}\\ &\,{{\mbox{THEN}}}\,\ \,{{\mbox{stimulus}}}\,\ \,{{\mbox{is}}}\,\ {Y}_{q}\ \,{{\mbox{with}}}\,\ D{S}_{q}\end{array}$$

where *q* is the pattern number, *NV*^*j*^ is the numeric brain activity value of fNIRS channel *j*, *CoL*^*j*^ is the conceptual label for the *j*th channel with *n* as the total number of channels, *Y*_*q*_ is the consequent stimulus class for the pattern, and *DS*_*q*_ is the dominance score associated with the *q*th pattern.

In the present work, a multivariate matrix is constructed by calculating the mean of the HbO2 signal for each of the 10 channels from time 4−7 s post-stimulus presentation for each trial. The rows in the multivariate matrix consist of all the trials with each entry in the two-dimensional matrix (for row (*i*) and column (*j*) being the average of the *j*th channel activity from time 4−7 s post-stimulus for the *i*th trial. Please see Fig. 1c that outlines the steps for the construction of a multivariate matrix.

In general, xMVPA inference mechanism consists of the following integral processes:

1. Brain activation concept definition;
2. Pattern dominance score evaluation;
3. Matching of data with the stimulus by the explainable pattern;
4. Learning of xMVPA:
  - Learning of conceptual labels;
  - Learning of patterns.

**Fig. 5:**
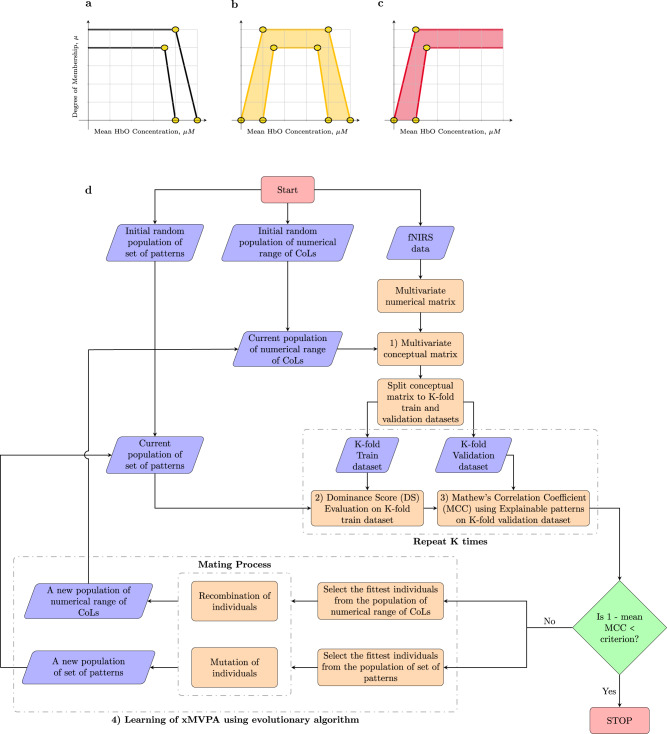
A flowchart outlining the construction of xMVPA. **a**−**d** An illustrative plot delineating the values to be learnt by the evolutionary algorithm for each of the conceptual labels (CoLs) associated with brain activity in a channel, with the corresponding degree of membership, *μ*, namely: *inactive* (white), *active* (amber), and *very active* (red). The values to be learnt for each CoLs definition are encircled in yellow. For CoL *inactive*, the values to be learnt are 4 as shown in (**a**), for CoL *active* are 8 as shown in (**b**), and for CoL *very active* the values to be learnt are 4 as shown in (**c**). **d** A flowchart depicting the steps for the construction of xMVPA. With an initial random population (of size 200) of the numerical range of CoLs, and an initial random population (of size 200) of a set of patterns, the input fNIRS data is converted into the conceptual multivariate matrix and split into five-fold train and validation datasets. The dominance score (DS) of the patterns is established using train datasets, and the validation datasets are used to determine the efficacy of the set of patterns based on MCC (Mathew’s correlation coefficient). The tolerance criterion, 1 × 10^−5^, for the evolutionary algorithm is compared with the 1 − mean MCC of the five-fold validation datasets to evaluate the performance of the set of patterns for correctly predicting the stimulus for un-labelled brain activity instances in the validation datasets. If the tolerance criterion is met, the xMVPA learning is complete else new populations, of size 200, of the numerical range of CoLs and set of patterns are found, and their DS and MCC on *k*-fold train and validation datasets are determined respectively and the process is repeated till the set tolerance criterion is achieved. The numbers in the boxes, 1)−4), refer to the steps outlined in the section “Methods”: a MVPA method via XAI (xMVPA) for the construction of the xMVPA.

The interlinks between the different processes of the xMVPA inference mechanism are delineated in a flowchart in Fig. 5d. A description of each of these processes is provided next.

In this work, evaluation of xMVPA is performed by splitting the observations transformed into the conceptual multivariate matrix into five mutually exclusive train and validation sets (viz. k-fold cross-validation). The patterns are initially generated at random with the maximum number of patterns in a given set to be 20, and the maximum number of channels (or antecedents) in a given pattern to be 3, i.e., a given pattern would outline interactions from a maximum of 3 channels/brain regions. The small number of patterns with short antecedents, ensures that a given set of patterns is comprehensive and easily interpretable^48,49^.

### Conceptualisation of brain activation levels

**Fig. 6:**
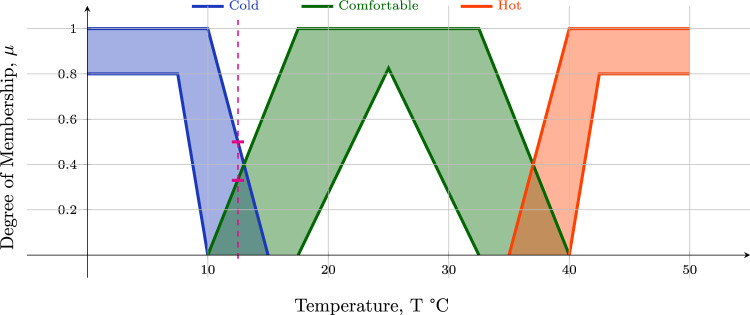
Membership functions of CoLs that integrate uncertainty handling in xMVPA. An illustrative plot to exemplify how conceptual labels (CoLs), which can be used to describe thermal comfort based on the room temperature (T ^∘^C), are characterised with uncertainty handling in xMVPA. Thermal comfort can be expressed with the CoLs *cold*, *comfortable,* and *hot* with an approximate degree of membership values, *μ*, obtained from ref. ^54^. As can be seen in the figure, the definition of CoLs is not necessarily mutually exclusive, i.e., a certain temperature can be represented using more than one CoL with varying degrees of membership. For example, the temperature of 12 ^∘^C has a degree of membership, *μ*, in the range of (0, 0.5) for *cold* and (0, 0.33) for *comfortable*. The derived ambiguity in the degree of membership ensures that uncertainty in the numeric data (or neuroimaging reading from fNIRS) is well retained upon transformation into a CoL.

The xMVPA works on a multivariate matrix that has elements characterised by CoLs. The numerical multivariate matrix formed by combining the data from all channels of interest is converted into a *conceptual* multivariate matrix. In the present work, the CoLs of *inactive*, *active*, and *very active* are used to represent the level of brain activity measured by a fNIRS channel. The capability of encoding uncertainty in the numerical range of each CoL makes the xMVPA particularly suitable for analysing infant neuroimaging data, which are typically characterised by high levels of inter-subject variability^50^. The CoLs also allow furnishing multivariate methods with activation-based analysis as well as information-based analysis^20^. An illustrative plot to exemplify how a CoL is characterised with uncertainty handling in xMVPA is shown in Fig. 6 with reference to thermal concepts.

The conversion of numeric data into CoLs is based on the *numerical range of values* represented by each of the CoLs. The shape of the degree of the membership functions for the CoLs is as outlined in Fig. 5a−c. The numeric values to be learnt for the definition of *inactive* and *very active* CoLs are 4 each, while 8 numeric values need to be optimised for the trapezium-shaped degree of membership function for *active*. The range of numeric values for each CoL are learnt using an evolutionary algorithm with more details as outlined in the section xMVPA learning from data.

A mathematical definition of the degree of the membership function is provided in section Supplementary Methods.

### Explainable patterns’ dominance score evaluation

Starting with an initial random set of patterns, the $\overline{{{{{{\mathrm{upper}}}}}}}$ and $\underline{{{{{{\mathrm{lower}}}}}}}$ bounds of the dominance score, ${\overline{DS}}_{q}$ and ${\underline{DS}}_{q}$, respectively, for each of the patterns *P*_*q*_ in the set are determined on a given k-fold training dataset as shown in eq. (4)^22^.

4 $${\overline{DS}}_{q}= \, {\overline{c}}_{q}\cdot {\overline{s}}_{q}\\ {\underline{DS}}_{q}= \, {\underline{c}}_{q}\cdot {\underline{s}}_{q}$$

where *q* is the pattern number, ${\overline{c}}_{q}$ and ${\underline{c}}_{q}$ is the upper and lower confidence score of the pattern *P*_*q*_ respectively, and ${\overline{s}}_{q}$ and ${\underline{s}}_{q}$ is the upper and lower support of the pattern *P*_*q*_, on a training dataset.

The confidence score, *c*_*q*_, of a pattern, *P*_*q*_, can be viewed as the possibility that a given data instance is an observation of this pattern, i.e., *c*_*q*_ is the likelihood of a given data instance to be a representative observation for the same stimulus as the pattern stimulus (consequent) *Y*_*q*_, given the data instance has matching interactions of brain regions as the rule *P*_*q*_, i.e., the same antecedents. More information on the pattern confidence is provided in eq. (3) in Supplementary Methods. The support, *s*_*q*_, of a given pattern is an indication of the coverage of the training dataset by the pattern. More information on the support is provided in eq. (4) in Supplementary Methods.

In this work, the conceptual multivariate data is split into five-fold disjoint training and validation datasets^51^ to ensure there is no bias in selecting the training and validation datasets. Also, please note that in the xMVPA inference mechanism, there is no information flow from the learning of patterns from one training fold to another training fold.

### Stimulus prediction

A set of optimal patterns, with corresponding dominance scores, *DS*_*q*_, are obtained using an evolutionary search (section xMVPA learning from data) guided by the results of a *k*-fold cross-validation (*k* = 5) procedure. The most possible stimulus for a given data instance, where a data instance is a row (*i*) in the validation dataset, is achieved by evaluating the association of the data instance with all the patterns (rule-based explanations). The stimulus-response of a data instance is predicted as the consequent of the pattern with the highest association degree, i.e., visual or auditory stimulus.

The stimulus for each data instance in the validation dataset, *x*_*i*_, is determined using the metric of association degree. The association degree, *h*_*q*_, of pattern *P*_*q*_ with each data instance in the validation dataset, *x*_*i*_, is computed as outlined in eq. (5).

5 $${\overline{h}}_{q}({x}_{i})= \, {\overline{w}}_{q}({x}_{i})\cdot {\overline{DS}}_{q}\\ {\underline{h}}_{q}({x}_{i})= \, {\underline{w}}_{q}({x}_{i})\cdot {\underline{DS}}_{q}\\ {h}_{q}({x}_{i})= \, \frac{{\overline{h}}_{q}({x}_{i})+{\underline{h}}_{q}({x}_{i})}{2}$$

where ${\overline{w}}_{q}$ and ${\underline{w}}_{q}$ are the upper and lower strengths of activating a pattern *P*_*q*_ for a data instance of the validation set *x*_*i*_. More information on the strengths of activation is provided in eq. (2) in Supplementary Methods.

In sum, a given validation data instance, *x*_*i*_, is classified as a response to the stimulus, *Y*_*q*_, corresponding to the pattern *P*_*q*_ with the maximum association degree with *x*_*i*_.

### xMVPA learning from data

The initial set of patterns used in the proposed xMVPA inference mechanism is randomly generated to ensure that there is no bias introduced in learning the set of patterns. An evolutionary genetic algorithm (GA) is integrated into the xMVPA inference mechanism to identify patterns that together give the best classification results.

Figure 5d outlines the steps undertaken to reveal an optimised set of patterns using a given dataset. All sets of patterns are learnt using *k*-fold cross-validation to establish the general ability of a given set of patterns.

Using an initial random set of patterns with a total of *Q* patterns, Mathew’s correlation coefficient (MCC) of the set of patterns is computed as MCC gives a more balanced measure of the quality of binary (two-class) classifications. It is computed as shown in eq. (6)^16^:

6 $${{{{{\mathrm{MCC}}}}}}=\frac{{{{{{\mathrm{TP}}}}}}\ \times \ {{{{{\mathrm{TN}}}}}}\ -\ {{{{{\mathrm{FP}}}}}}\ \times {{{{{\mathrm{FN}}}}}}}{\sqrt{({{{{{\mathrm{TP}}}}}}\ +\ {{{{{\mathrm{FP}}}}}})({{{{{\mathrm{TP}}}}}}\ +\ {{{{{\mathrm{FN}}}}}})({{{{{\mathrm{TN}}}}}}\ +\ {{{{{\mathrm{FP}}}}}})({{{{{\mathrm{TN}}}}}}\ +\ {{{{{\mathrm{FN}}}}}})}}$$

where TP, TN, FP, and FN are as defined in the confusion matrix in Fig. 3a.

The cost of the set of patterns is computed as 1 − the mean of the MCCs of all *k*-fold validation datasets. The GA then compares the cost of the set of patterns with a pre-defined tolerance criterion. If the cost is greater than the tolerance of GA, the GA then populates a new set of patterns, and the cycle is repeated till the tolerance criterion of the GA is met as outlined in Fig. 5d. More details on the GA are provided in subsection 1.5 in Supplementary Methods.

To maximise the model interpretability, the total number of patterns to be learnt by xMVPA system is set at 20 patterns, with a maximum of three channels interactions in a given pattern (as three-point messages are the recommended standard for science communications^49^). The evolutionary system^52^ will aim to maximise prediction accuracy while using a maximum of 20 patterns, where each pattern consists of a maximum three antecedents. This renders the total number of variables, to be optimised for pattern learning, by GA to be: total number of patterns (20) * maximum number of channels (3) and CoL for each chosen channel (3: inactive, active, or very high) and the corresponding stimulus class for each pattern (1) = 20*(3 + 3 + 1) = 140 variables.

The number of parameters to be learned for CoLs definition is the lower and upper numeric values for each concept. For a given channel, the number of variables that need to be learned for the channel’s equivalent CoLs numeric range is 16 (4 for inactive (IA) as shown in Fig. 5a, 8 for active(A) as shown in Fig. 5b, and 4 for very active (VA) as shown in Fig. 5c). Hence, in this work, for ten channels, the total number of variables to be optimised for CoLs numeric range are 16 $*$ 10 = 160.

Hence, the grand total of variables to be learnt by the GA is 140 + 160 = 300 variables. The structure of each phenotype is delineated in eq. (7). The population size of GA, i.e., the number of feasible solutions, is set at 200, with selection done using *tournament*, and the GA tolerance is set at 1 $*$ 10^−5^.

7 $${\rho }^{b}= \, \left\{{\phi }_{1}^{1},{\phi }_{2}^{1},{\phi }_{3}^{1},{\lambda }_{1}^{1},{\lambda }_{2}^{1},{\lambda }_{3}^{1},{\gamma }^{j,1},...,\right.\\ \, {\phi }_{1}^{Q},{\phi }_{2}^{Q},{\phi }_{3}^{Q},{\lambda }_{1}^{Q},{\lambda }_{2}^{Q},{\lambda }_{3}^{Q},{\gamma }^{j,Q},...,\\ \, {\delta }_{{IA}^{j}}^{1},...,{\delta }_{{IA}^{j}}^{4},{\delta }_{{A}^{j}}^{1},...,{\delta }_{{A}^{j}}^{8},{\delta }_{{VA}^{j}}^{1},...,{\delta }_{{VA}^{j}}^{4},...,\\ \, \left.{\delta }_{{IA}^{n}}^{1},...,{\delta }_{{IA}^{n}}^{4},{\delta }_{{A}^{n}}^{1},...,{\delta }_{{A}^{n}}^{8},{\delta }_{{VA}^{n}}^{1},...,{\delta }_{{VA}^{n}}^{4}\right\}^{\prime}$$

where *ρ*^*b*^ is the phenotype of an individual *b* (a potential solution) for the GA for a total of Q patterns. Each *ϕ* denotes a particular channel, and each *λ* represents the corresponding CoLs associated with each channel. These chromosomes form the antecedent of a pattern. The consequent of this pattern is denoted as *γ*. The *δ* represents the numeric values for the range of each of the CoLs of all the *n* Chs. In particular, ${\delta }_{{{{{{{\mathrm{CoL}}}}}}}^{j}}^{{{{{{\mathrm{NV}}}}}}}$, subscript CoL denotes the value of concept that can be *inactive*: IA, *active*: A, and *very active*: VA, along with the associated channel number *j* and the numeric value (NV) in the superscript: 4 NVs for *inactive* and *very active*, and 8 NVs for *active*.

### Statistics and reproducibility

A total of 19 babies’ data is included in the analysis, with multiple trials per baby, amounting to 524 trials. Experimental control and signal assessment were performed to avoid any possible noise artifacts or covariates in our data^19,53^. The evaluation of xMVPA is performed by splitting the observations transformed into the conceptual multivariate matrix into five mutually-exclusive train and validation sets (viz. *k*-fold cross-validation). The statistical performance measures that attain statistical significance over the decoding results of a baseline classifier with 5,000 randomly permuted stimulus labels are reported in Fig. 3 and denoted with asterisks (*). Moreover, additional statistical values are reported in Supplementary Table 4.

### Reporting summary

Further information on research design is available in the Nature Research Reporting Summary linked to this article.

## Supplementary information

Supplementary Information

Reporting Summary

## Data Availability

The data from the analysis of this paper is publicly available at the Princeton Data Repository: http://arks.princeton.edu/ark:/88435/dsp01xs55mf543. Data used in section 2.2 of the Supplementary File is available at http://bnci-horizon-2020.eu/database/data-sets. Data used to generate Figs. 2−4 from the main article and Figs. 1−3 of the Supplementary Material are provided with the paper.

## Code availability

An executable version of xMVPA is publicly available on GitHub (https://github.com/jandreu/xMPVA) and Zenodo (10.5281/zenodo.4644294), for other developmental scientists to use it. The source code of xMVPA is available through sharing agreement upon reasonable request to the corresponding author.
