# Supplementary material for: Explainable artificial intelligence based analysis for interpreting infant fNIRS data in developmental cognitive neuroscience
Source: Commun Biol. 2021 Sep 15;4:1077. doi: 10.1038/s42003-021-02534-y (PMC8443619; doi:10.1038/s42003-021-02534-y)
Supplement: Supplementary file 1 — Supplementary Information [file 42003_2021_2534_MOESM1_ESM.pdf]

Explainable Artificial Intelligence Based Analysis for Interpreting Infant fNIRS data in Developmental Cognitive Neuroscience

Supplementary File

Javier Andreu-Perez*[1], Lauren L. Emberson[2], Mehrin Kiani[1], Maria Laura Filippetti[3], Hani Hagras[1], and Silvia Rigato[3]

[*]Corresponding author email: javier.andreu@essex.ac.uk

[1]Centre for Computational Intelligence, University of Essex, Colchester, CO4 3SQ, United Kingdom

[2]Department of Psychology, Princeton University, NJ 08544, United States of America

[3]Centre for Brain Science, Department of Psychology, University of Essex, Colchester, CO4 3SQ, United Kingdom

1 Supplementary Methods

1.1 Degree of membership function

In this work, the CoLs are defined using interval type-2 fuzzy concepts $\tilde{\mathrm{A}}$ hagras2018xai . In mathematical notation, $\tilde{\mathrm{A}}$ can be written as follows in eq. (1).

$$\begin{aligned} \tilde{\mathrm{A}} = \{(x, u, 1) | \forall x \in X, \\ \forall u \in [\underline{\mu}_{\tilde{\mathrm{A}}}(x), \overline{\mu}_{\tilde{\mathrm{A}}}(x)] \subseteq [0, 1]\} \end{aligned} \tag{1}$$

where $\mu_{\tilde{\mathrm{A}}}$ represent the membership degree function of interval type-2 fuzzy concept $\tilde{\mathrm{A}}$.

Fig. 6 shows an illustrative plot for interval type-2 fuzzy concepts of thermal comfort: *Cold*, *Comfortable*, and *Hot*. The membership degree for temperature of 12 °C, in Fig. 6, falls in the interval type-2 fuzzy concept of $\tilde{Cold}$ with lower and upper membership degree function values as: $\underline{\mu}_{\tilde{\mathrm{C}}\mathrm{old}}(12) = 0$ and $\overline{\mu}_{\tilde{\mathrm{C}}\mathrm{old}}(12) = 0.5$. Likewise, the membership degree for temperature of 12 °C falls in the interval type-2 fuzzy sets of $\tilde{Comfortable}$ with lower and upper membership degree values as: $\underline{\mu}_{\tilde{\mathrm{C}}\mathrm{omfortable}}(12) = 0$ and $\overline{\mu}_{\tilde{\mathrm{C}}\mathrm{omfortable}}(12) = 0.33$.

1.2 Strength of activation

The strength of activation, $w_q(x_i)$, of pattern, P_q, for a data instance, x_i^k, is a measure of the degree of match between the pattern and the data instance. It is computed as outlined in (2).

$$
\begin{aligned}
\overline{w}_q(x_i) &= \prod_{k=1}^{a} \overline{\mu}_{\tilde{A}^k}(x_i^k) \\
\underline{w}_q(x_i) &= \prod_{k=1}^{a} \underline{\mu}_{\tilde{A}^k}(x_i^k)
\end{aligned} \tag{2}
$$

where $a \subseteq \{1, ..., n\}$, is the total number of antecedents.

1.3 Pattern confidence

The confidence, c_q, of a pattern, P_q, is an indication of the likelihood of a pattern correctly classifying a data instance. It is computed using

$$
\begin{aligned}
\overline{c}_q(A_q \Rightarrow Y_q) &= \frac{\sum_{x_{i,t} \in (A_q \Rightarrow Y_q)} \overline{w}_q(x_{i,t})}{\sum_{q=1, x_{i,t} \in A_q}^{Q} \overline{w}_q(x_{i,t})} \\
\underline{c}_q(A_q \Rightarrow Y_q) &= \frac{\sum_{x_{i,t} \in (A_q \Rightarrow Y_q)} \underline{w}_q(x_{i,t})}{\sum_{q=1, x_{i,t} \in A_q}^{Q} \underline{w}_q(x_{i,t})}
\end{aligned} \tag{3}
$$

where $\overline{w}_q(x_{i,t})$ and $\underline{w}_q(x_{i,t})$ are the upper and lower strengths of activation, as outlined in eq. (2) in Appendix B, for pattern P_q on a data instance $x_{i,t}$ belonging to training dataset t, A_q is the antecedent, and Y_q is the consequent of the pattern P_q (previously defined in section Methods: A MVPA method via XAI (xMVPA)).

1.4 Pattern support

The support, s_q, of a pattern, P_q, is an indication of the coverage of training dataset by the pattern. It is computed using (4).

$$
\begin{aligned}
\overline{s}_q(A_q \Rightarrow Y_q) &= \frac{\sum_{x_{i,t} \in (A_q \Rightarrow Y_q)} \overline{w}_q(x_{i,t})}{Q} \\
\underline{s}_q(A_q \Rightarrow Y_q) &= \frac{\sum_{x_{i,t} \in (A_q \Rightarrow Y_q)} \underline{w}_q(x_{i,t})}{Q}
\end{aligned} \tag{4}
$$

where $\overline{w}_q(x_{i,t})$ and $\underline{w}_q(x_{i,t})$ are the upper and lower strengths of activation for pattern P_q on a data instance $x_{i,t}$ in a training dataset, A_q is the antecedent, and Y_q is the consequent of the pattern P_q (previously defined in section Methods: A MVPA method via XAI (xMVPA) in the manuscript) with Q as the total number of patterns (as defined in section xMVPA learning from data in the manuscript).

1.5 Genetic algorithm (GA)

Genetic algorithms (GA) are a type of evolutionary algorithm[1] that are based on the *survival of the fittest* phenomenon from Darwin's evolutionary theory. The 'survival of fittest' idea states that given limited resources for a population of individuals within some environment, a competition for those resources causes a natural selection of the individuals in that population and eventually only the *fittest* individuals *survive*, i.e. gain access to the limited resources. Consequently, the population of the individuals that survive are the best of the possible individuals.

In the present work, individual solutions (which comprise of the set of patterns, and the numeric range of CoLs, see eq. (7)) cost (1 - the mean of the resultant MCCs from cross-validation) is compared against a set tolerance criterion (see Fig. 5). The solutions that have the best fitness values become the 'parents' for the next generation (offsprings) of the 'solutions'. The next generation of solutions is found by incorporating novelty using recombination and/or mutation in the parent solutions. Recombination is an operator that is applied on two or more parents to produce the offsprings whereas mutation is applied to one parent and results in a new altered/mutated offspring. In this way, the application of recombination and mutation generates a novel generation of the solutions. In turn, these newly generated solutions are evaluated against the tolerance criterion and given fitness score. If the fitness score is less than the tolerance criterion the search for the optimal solution is stopped, else the process is iterated until a solution with sufficient quality (i.e. meets the tolerance criterion) is found or an iteration limit is reached.

2 Supplementary Results

2.1 Exemplar case study of xMVPA with Deoxygenated Haemoglobin

In this section, we present the results of the xMVPA inference mechanism on the deoxygenated haemoglobin (deoxy-Hb) signals obtained from Emberson et al.[2]. The xMVPA is applied on the multivariate matrix formed by calculating the mean of the deoxy-Hb signals from each of the 10 channels in the time-window 4-7s, following stimulus presentation, for each trial. Please note that the construction of the multivariate matrix and the xMVPA parameters are identical for both oxygenated haemoglobin (oxy-Hb) and deoxy-Hb signals. The xMVPA results for oxy-Hb are presented in the main manuscript.

The evaluation of xMVPA on the multivariate matrix from deoxy-Hb signals gives an average classification accuracy of 64.88% with a standard deviation of 4.81%. The eight patterns provided by xMVPA that outline the brain regions' activation and interaction for processing visual and auditory information for deoxy-Hb signals are given below:

Pattern P_1 : IF Ch2 is *Active* AND Ch3 is *Very Active*
THEN stimulus is *Visual* with dominance score 0.02

Pattern P_2 : IF Ch5 is *Very Active* AND Ch8 is *Very Active*
THEN stimulus is *Visual* with dominance score 0.01

Pattern P_3 : IF Ch2 is *Very Active* AND Ch5 is *Very Active*
THEN stimulus is *Auditory* with dominance score 0.67

Pattern P_4 : IF Ch4 is *Very Active* AND Ch7 is *Very Active*
THEN stimulus is *Auditory* with dominance score 0.08

Pattern P_5 : IF Ch2 is *Very Active* AND Ch9 is *Active*
THEN stimulus is *Auditory* with dominance score 0.08

Pattern P_6 : IF Ch1 is *Inactive* AND Ch9 is *Active*
THEN stimulus is *Auditory* with dominance score 0.06

Pattern P_7 : IF Ch2 is *Inactive* AND Ch9 is *Active*
THEN stimulus is *Auditory* with dominance score 0.03

Pattern P_8 : IF Ch1 is *Active* AND Ch7 is *Active* AND Ch9 is *Very Active*
THEN stimulus is *Auditory* with dominance score 0.02

where dominance score (DS) is in the range [0,1]. The greater the value of DS the more informative that pattern is with DS=0 being the least informative pattern.

A total of two patterns, P_1 and P_2, have been identified by xMVPA for the processing of visual information from the deoxy-Hb signals. P_1 delineates the contributions of only occipital channels, i.e. channels 2 and 3, whereas P_2 identifies the contributions between channel 5 (temporal cortex) and channel 8 (prefrontal cortex). However, none of the channels from the occipital and prefrontal cortex have been found engaged by the xMVPA for visual processing. Moreover, the dominance score (DS) of the patterns is almost negligible, i.e. 0.02 and 0.01 for P_1 and P_2 respectively.

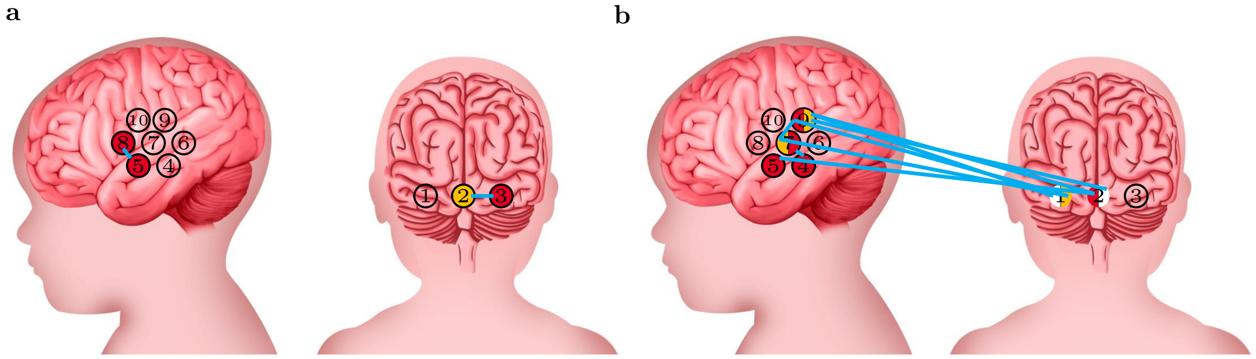

Supplemetary Figure 1: An illustration of the patterns (cyan) identified by the xMVPA, using deoxy-Hb signals, delineate the contributions between brain regions evoked by **a** visual and **b** auditory stimuli. It is important to note here that most of the patterns found by the xMVPA from deoxy-Hb signals are unreliable with dominance scores (DS) less than 0.05. The colour of the channels denote their level of activity: Inactive (white), Active (amber) and Very Active (red) and uncoloured for channels that do not belong to any pattern.

For the auditory processing, the xMVPA found six patterns: P_3 - P_8. However, only P_3 is a relevant pattern with a DS of 0.67. P_3 uncovers the contributions of occipital and temporal channels as it outlines both channel 2 (occipital cortex) and channel 5 (temporal cortex) to be very active. The remaining patterns, P_4 - P_8, for auditory processing are not as supported as P_3, with P_4 delineating contributions within the temporal cortex (channel 4 and channel 7) and P_5 - P_8 outlining contributions between the occipital and temporal cortices. Unlike the patterns found for visual processing, none of the patterns for the auditory processing outline the contributions from the prefrontal cortex.

An illustration of the patterns for both visual and auditory processingis shown in Supplementary Fig. 1.

An illustration of the cortical network formed using the xMVPA patterns obtained from deoxy-Hb signals of six-months-old infants for the processing of auditory stimulus is shown in Supplementary Fig. 2. There is no prominent cortical network for visual processing using deoxy-Hb signals since the two xMVPA patterns P_1 and P_2, for visual processing, have almost negligible DS.

As such, no direct comparison of visual cortical networks formed from the xMVPA patterns using the oxy-Hb and deoxy-Hb is possible since no prominent cortical network is uncovered for the visual processing using deoxy-Hb signals. A comparison for the cortical network for the auditory processing, for oxy-Hb and deoxy-Hb, reveals a notable absence of prefrontal cortex for the deoxy-Hb cortical network.

Although we have presented a preliminary comparison of the cortical networks formed for the processing of oxy-Hb and deoxy-Hb, it is important to note that most developmental studies with fNIRS do not investigate the deoxy-Hb signals because of their low signal-to-noise ratio, as well as inconsistent response in infants[3,4]. We further validated the inefficacy of the deoxy-Hb signals for the six-month-old infants data by comparing the decoding accuracy of oxy-Hb signals with deoxy-Hb signals using correlation based MVPA and other state-of-the-art classifiers, and our proposed method of xMVPA in Supplementary Table 1. As can be readily appreciated from the decoding accuracy, values of xMVPA reported in Supplementary Table 1 are indeed similar to or better than the ones obtained with opaque box methods, while rendering insightful explainability on the decoding patterns. Nevertheless, mostly low dominance patterns were present with deoxy-Hb. This corroborates what is reported in DCN literature[3,4] that decoding of oxy-Hb signals is more consistent and informative than deoxy-Hb signals.

Given the unreliable deoxy-Hb signals, a lack of infant studies investigating deoxy-Hb signals in the literature, and mostly under-supported xMVPA patterns on account of low DS, we cannot discuss the implications of the xMVPA patterns obtained using deoxy-Hb signals.

Supplementary Table 1: A comparison of average decoding accuracy (avg.) with standard deviation (SD) using oxy-Hb and deoxy-Hb data from the earlier work of Emberson et al.[2]. The accuracies with the two dimensions are reported for the correlation based MVPA (as is done in the earlier study[2]), Support Vector Machine (SVM), Random Forest (RF), Multi-layer perceptron (MLP) and the proposed method eXplainable MVPA (xMVPA).

**Haemoglobin**	**MVPA[2]**		**SVM**		**RF**		**MLP**		**xMVPA**	
	Avg.	*SD*	*Avg.*	*SD*	*Avg.*	*SD*	*Avg.*	*SD*	*Avg.*	*SD*
oxy-Hb	66.67	17.45 %	69.87	1.17	67.47	5.32	68.36	3.22	67.69	3.52%
deoxy-Hb	33.98	16.57 %	61.65	3.29	60.76	2.47	57.72	3.21	64.88	4.81%

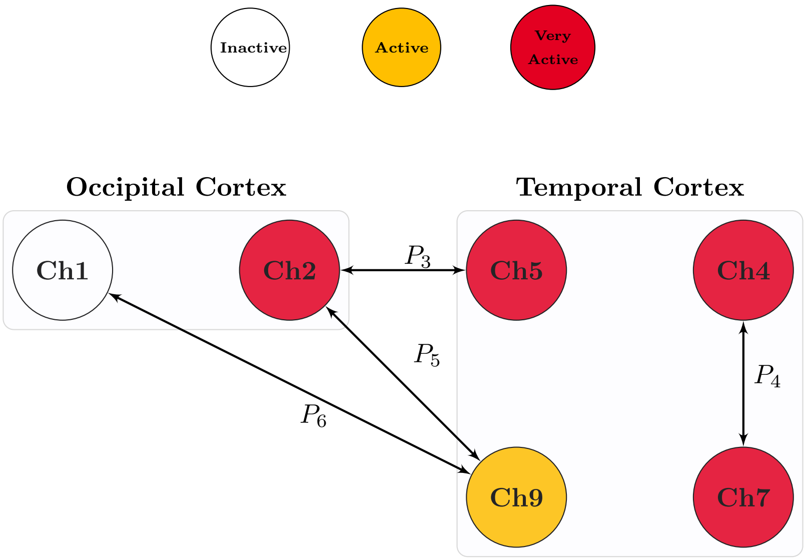

Supplementary Figure 2: An illustration of the cortical network formed for non-speech auditory processing in six-month-old infants, based on the patterns P_3 to P_6 (P_7 and P_8 are not shown because of negligible dominance score (DS)) revealed by the xMVPA inference mechanism using deoxygenated haemoglobin (deoxy-Hb) signals. The proposed model consists of the temporal cortex and occipital cortex with no involvement of the prefrontal cortex. The colour of the channel's (Ch) circle is based on its activity level: Inactive (white), Active (amber), and Very Active (red). Please note that the patterns outlined for the visual processing have very low dominance score (DS), and owing to their insignificance, no cortical network illustration is shown for visual processing.

2.2 Exemplar case study of xMPVA in Adult fNIRS Dataset on Mental Arithmetic

In this section, we exemplify an application of xMVPA on an adult fNIRS dataset which is made publicly available by Bauernfeind *et al.*[5]. In the aforementioned study, a total of 8 adults brain activity were recorded whilst they are performing a cognitive task (i.e. mental arithmetic) and when they are at rest (i.e. not performing any task). For more information on the data collection, and preprocessing stages please see the earlier study[5].

The earlier study[5] aimed at detecting the 'antagnositic pattern' (AP) which is an increase in dorsolateral prefrontal cortex (DLPFC) and a simultaneous decrease (lower activity) in anterior prefrontal cortex (APFC)[6] using oxy-Hb signals during a mental arithmetic task. The anatomical location of the channels used in the original study are APFC: 46, 47, 58; left DLPFC: 18, 28, 29; right DLPFC: 23, 24[5]. The earlier work carried an exhaustive search to identify the times post stimulus and channels for mental arithmetic and rest that gave the best classification accuracy between the two classes, i.e. mental arithmetic and rest. The times post stimulus presentation that gave the best classification results in the earlier work are as noted in Supplementary Table 2.

In the present work, we used the same values of times for both mental arithmetic and rest to construct the multivariate matrix using oxy-Hb signals however the channels that would give the best classification accuracy is chosen by the xMVPA. The evaluation of xMVPA on the multivariate matrix from oxy-Hb signals is performed in the same manner as used by the original study[5]. The accuracy obtained for each subject are listed in Supplementary Table 2.

The two most important patterns found for each of the class, i.e. mental arithmetic and rest, for each subject are listed in Supplementary Table 3. The xMVPA has been able to identify static (i.e. time resolved

Supplementary Table 2: Decoding accuracy (Acc.) for each of the 8 subjects (Sub.) independently for mental arithmetic (MA) task vs. rest using xMVPA and original study's linear discriminant analysis. The time (t) in seconds post MA stimulus identified in the original study[5] has been used for xMVPA as well. For the sake of comparison, we used the same evaluation criteria as in[5].

**Sub.**	$\mathbf{t}_{MA}$	$\mathbf{t}_{Rest}$	**Acc.[5]**	**xMVPA Acc.**
1	10	29	68.75%	75.0%
2	13	30	87.50%	100.0%
3	12	29	75.00%	100.0%
4	14	28	73.50%	93.75%
5	13	26	81.25%	81.25%
6	10	26	68.75%	87.50%
7	10	29	87.50%	87.50%
8	12	26	81.25%	87.50%

for the times listed in Supplementary Table 2) AP for across subjects dataset, as well as for within subjects for all subjects except subject no. 6. A decrease in performance for subject no. 6 was also found in the original study and xMPVA also did not determine any APs between the DLPFC and APFC, in-contrary to the other subjects. This may not be related to the prowess of xMVPA in finding meaningful patterns, but a result of a particular issue reported as *BCI illiteracy*[7] in which 20% of the subjects are not able to intentionally elicit the requested brain response or lack the ability to sustain attention in the task, and therefore the characteristic patterns are not present.

Our results are congruent with the ones reported by Baurnefield et al.[5], which hypothesised that the AP pattern is prominent in adult subjects performing a mental arithmetic task. The advantage of using xMVPA is that it is able to shed light on the subjects' underlying neural patterns, which was not possible with the opaque black-box models previously employed with this data. The group analysis reported that AP was a determinant pattern to decode the mental versus rest task. Moreover, we can also see that inter-subject differences do not affect xMVPA in discovering the specific AP patterns for each subject, regardless of the magnitude of their activation response (see Supplementary Table 3). Besides expressing patterns as propositional logic statements, another inherent advantage of xMPVA is that it can represent fNIRS responses as conceptual linguistic sets (CoLs), which helps to assimilate inter-subject variabilities in the analysis. As shown in Supplementary Table 2, decoding accuracy was similar to or better than the black-box model used in Baurnefield et al.[5]. Furthermore, the greater transparency of xMVPA permits revealing which subjects did not elicit the expected AP response, elucidating the reasons behind the low decoding accuracy exhibited in the previous work for some subjects. An illustration of the AP for each subject is displayed in Supplementary Fig. 3.

Supplementary Table 3: The most relevant patterns for each subject (Sub.), i.e. patterns with dominance score (DS) > 0.05, for each class: Mental arithmetic (MA) and Rest with DS are presented. The pattern found by xMVPA which can be representative of the time resolved antagonistic pattern (AP) is highlighted in blue. The AP is an increase in dorsolateral prefrontal cortex (DLPFC) and a simultaneous decrease in anterior prefrontal cortex (APFC)[5]. In the original study the channels (ch) in left DLPFC: 18, 28, and 29 and in right DLPFC are: 23, 24. The channels in APFC are: 46, 47, and 48.

Sub.	Rule	DS	
1	IF ch 29 is active and ch 46 is inactive THEN MA	0.63	← AP
	IF ch 18 is active and ch 46 is active and ch 48 is active THEN MA	0.40	
	IF ch 46 is active and ch 48 is active THEN Rest	0.46	
	IF ch 29 is very active and ch 48 is active THEN Rest	0.16	
2	IF ch 28 is very active and ch 48 is active THEN MA	0.10	← AP
	IF ch 28 is very active and ch 29 is very active THEN MA	0.04	
	IF ch 24 is inactive THEN Rest	0.18	
	IF ch 46 is very active and ch 47 is very active THEN Rest	0.18	
3	IF ch 29 is active THEN MA	4.32	
	IF ch 29 is active and ch 47 is inactive THEN MA	1.00	← AP
	IF ch 46 is active and ch 47 is active THEN Rest	2.36	
	IF ch 24 is inactive and ch 29 is active and ch 46 is active THEN Rest	0.10	
4	IF ch 18 is active and ch 46 is inactive then MA	0.13	← AP
	IF ch 18 is inactive and ch 48 is medium then MA	0.03	
	IF ch 29 is active and ch 46 is very active then Rest	0.13	
	IF ch 28 is inactive and ch 29 is inactive and ch 47 is very active then Rest	0.09	
5	IF ch 29 is very active and ch 46 is active THEN MA	3.44	← AP
	IF ch 24 is inactive and ch 29 is very active THEN MA	2.89	
	IF ch 47 is active THEN Rest	0.42	
	IF ch 46 is active and ch 47 is active THEN Rest	0.41	
6	IF ch 29 is active and ch 47 is active THEN MA	0.29	
	IF ch 24 is active THEN MA	0.19	
	IF ch 46 is active and ch 47 is active THEN Rest	0.54	
	IF ch 24 is active and ch 29 is active and ch 46 is medium THEN Rest	0.43	
7	IF ch 28 is active and ch 46 is inactive and ch 48 is inactive THEN MA	0.14	← AP
	IF ch 24 is active and ch 46 is active and ch 48 is active THEN MA	0.06	
	IF ch 28 is active and ch 29 is active and ch 46 is active THEN Rest	0.24	
	IF ch 24 is active and ch 29 is active and ch 47 is inactive THEN Rest	0.02	
8	IF ch 18 is very active and ch 28 is active THEN MA	0.18	← AP
	IF ch 28 is active and ch 29 is active and ch 47 is active THEN MA	0.04	
	IF ch 28 is inactive and ch 29 is inactive THEN Rest	0.11	
	IF ch 24 is active and ch 28 is inactive and ch 48 is medium THEN Rest	0.07	
1 - 8	IF ch 46 is inactive and ch 47 is active THEN MA	0.12	
	IF ch 24 is active and ch 28 is active and ch 47 is inactive THEN MA	0.05	← AP
	IF ch 24 is very active and ch 47 is active THEN Rest	0.48	
	IF ch 18 is inactive and ch 24 is inactive and ch 47 is inactive THEN Rest	0.18	

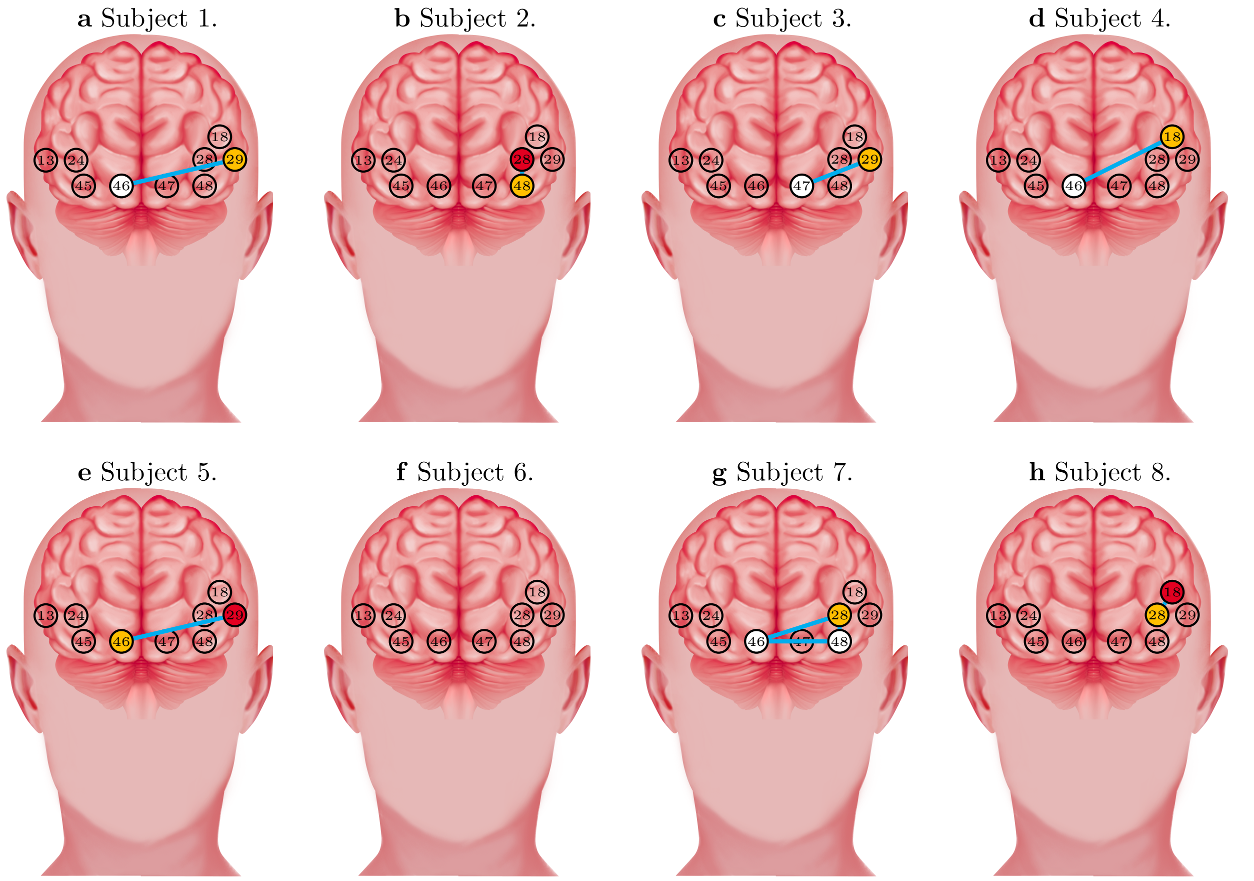

Supplementary Figure 3: An illustration of the time-resolved antagnostic pattern (AP) discovered by xMVPA for subjects 1-8 is shown with cyan solid line. The AP is an increase in dorsolateral prefrontal cortex (DLPFC) and a simultaneous decrease in anterior prefrontal cortex (APFC)[5]. The channels are denoted with circles. The colour of the circle represents the respective channel's activity with white denoting inactive channel, amber active, red representing a very active channel, and uncoloured channels that do not belong to any pattern. The channels 45, 46, 47 and 48 are in APFC with channels 28, 29, and 18 in right DLPFC and channels 13 and 24 in left DLPFC.

3 Detail of decoding performance results in Fig. 3 (main manuscript)

In Supplementary Table 4 is provided a full breakdown of the values of the performance metrics and statistics reported in section Results, and graphically illustrated in Fig. 3, of the manuscript.

Supplementary Table 4: In this table is presented a full description of the statistics reported in Fig. 3, including the statistics value and lower and upper confidence intervals (*Conf. Interval*). The test used to assess the reliability of the performance statistics was Bootstrapping analysis based on 5,000 simulations using a dummy (stratified) classifier as baseline performance[8]. All significance test here presented are one tailed in the positive direction of the performance metric.

**Perf. metric**	**Statistic**	**xMVPA**	**SVM**	**RF**	**MLP**	*Conf. Interval*
**Accuracy**	Avg.	67.69%	69.87%	67.47%	68.35%	[40.38% , 59.62%]
	SD	3.52%	1.17%	5.32%	3.23%	
	p-value	< 0.0001	< 0.0001	< 0.0001	< 0.0001	
	p < 0.05	True	True	True	True	
	p < 0.01	True	True	True	True	
**PPV**	Avg.	64.38%	69.14%	65.5%	68.71%	[37.88% , 61.40%]
	SD	5.8%	4.15%	4.11%	11.62%	
	p-value	0.0002	< 0.0001	0.0002	< 0.0001	
	p < 0.05	True	True	True	True	
	p < 0.01	True	True	True	True	
**NPV**	Avg.	73.95%	69.17%	66.4%	65.26%	[34.38% , 66.67%]
	SD	4.33%	4.91%	5.46%	3.59%	
	p-value	0.0002	0.0024	0.0074	0.0124	
	p < 0.05	True	True	True	True	
	p < 0.01	True	True	True	False	
**F-score**	Avg.	67.14%	69.73%	67.11%	67.87%	[39.58% , 58.56%]
	SD	3.3%	1.21%	5.53%	3.54%	
	p-value	< 0.0001	< 0.0001	< 0.001	< 0.0001	
	p < 0.05	True	True	True	True	
	p < 0.01	True	True	True	True	
**FPR**	Avg.	45.2%	58.83%	62.61%	50.45%	[39.68% , 95.35%]
	SD	12.15%	5.51%	7.91%	13.82%	
	p-value	0.0032	0.2383	0.2721	0.0096	
	p < 0.05	True	False	False	True	
	p < 0.01	True	False	False	True	
**FNR**	Avg.	20.38%	72.66%	70.87%	83.26%	[20.37% , 62.16%]
	SD	6.63%	2.98%	3.32%	16.12%	
	p-value	0.0014	0.9924	0.9896	0.9888	
	p < 0.05	True	False	False	False	
	p < 0.01	True	False	False	False	

Supplementary References

1. Herrera, F. Genetic fuzzy systems: taxonomy, current research trends and prospects. *Evolutionary Intelligence* **1,** 27–46 (2008).
2. Emberson, L. L., Zinszer, B. D., Raizada, R. D. S. & Aslin, R. N. Decoding the infant mind: Multivariate pattern analysis (MVPA) using fNIRS. *PLoS ONE* **12,** e0172500 (2017).
3. Lloyd-Fox, S., Blasi, A. & Elwell, C. E. Illuminating the Developing Brain: The Past, Present and Future of Functional Near Infrared Spectroscopy. *Neurosci Biobehav Rev.* **34,** 269–84 (2010).
4. Hespos, S. J., Ferry, A. L., Cannistraci, C. J., Gore, J. & Park, S. in *Imaging the Brain with Optical Methods* 159–176 (Springer, New York, 2010).
5. Bauernfeind, G., Scherer, R., Pfurtscheller, G. & Neuper, C. Single-trial classification of antagonistic oxyhemoglobin responses during mental arithmetic. *Medical and biological engineering and computing* **49,** 979–984 (2011).
6. Pfurtscheller, G., Bauernfeind, G., Wriessnegger, S. C. & Neuper, C. Focal frontal (de) oxyhemoglobin responses during simple arithmetic. *International Journal of Psychophysiology* **76,** 186–192 (2010).
7. Allison, B. Z. & Neuper, C. in *Brain-computer interfaces* 35–54 (Springer, London, 2010).
8. Pedregosa, F. *et al.* Scikit-learn: Machine Learning in Python. *Journal of Machine Learning Research* **12,** 2825–2830 (2011).
