# Supplementary material for: Explainable artificial intelligence based analysis for interpreting infant fNIRS data in developmental cognitive neuroscience
Source: Commun Biol. 2021 Sep 15;4:1077. doi: 10.1038/s42003-021-02534-y (PMC8443619; doi:10.1038/s42003-021-02534-y)
Supplement: Supplementary file 2 — Reporting Summary [file 42003_2021_2534_MOESM2_ESM.pdf]

nature research

Corresponding author(s): Javier Andreu-Perez

Last updated by author(s): Apr 27, 2021

Reporting Summary

Nature Research wishes to improve the reproducibility of the work that we publish. This form provides structure for consistency and transparency in reporting. For further information on Nature Research policies, see our Editorial Policies and the Editorial Policy Checklist.

Statistics

For all statistical analyses, confirm that the following items are present in the figure legend, table legend, main text, or Methods section.

n/a	Confirmed	
☐	☒	The exact sample size (n) for each experimental group/condition, given as a discrete number and unit of measurement
☐	☒	A statement on whether measurements were taken from distinct samples or whether the same sample was measured repeatedly
☐	☒	The statistical test(s) used AND whether they are one- or two-sided<br>*Only common tests should be described solely by name; describe more complex techniques in the Methods section.*
☐	☒	A description of all covariates tested
☐	☒	A description of any assumptions or corrections, such as tests of normality and adjustment for multiple comparisons
☐	☒	A full description of the statistical parameters including central tendency (e.g. means) or other basic estimates (e.g. regression coefficient) AND variation (e.g. standard deviation) or associated estimates of uncertainty (e.g. confidence intervals)
☐	☒	For null hypothesis testing, the test statistic (e.g. F, t, r) with confidence intervals, effect sizes, degrees of freedom and P value noted<br>*Give P values as exact values whenever suitable.*
☒	☐	For Bayesian analysis, information on the choice of priors and Markov chain Monte Carlo settings
☒	☐	For hierarchical and complex designs, identification of the appropriate level for tests and full reporting of outcomes
☒	☐	Estimates of effect sizes (e.g. Cohen's d, Pearson's r), indicating how they were calculated

Our web collection on statistics for biologists contains articles on many of the points above.

Software and code

Policy information about availability of computer code

Data collection	fNIRS recordings were collected at 10 Hz (every 100 ms). Marks were presented from MATLAB using a serial port on the stimulus presentation computer to the Hitachi ETG-4000 using standard methods. Marks were sent for the start and end of each presentation type for the given experiment (e.g., blocks of AV trials, single AV trials, and single omission trials). The raw data were exported from the Hitachi ETG-4000 to MATLAB using Hitachi MATLAB interoperability drivers and subsequent analyses with HomER 1 (Hemodynamic Evoked Response NIRS data analysis GUI, version 4.0.0) using the default preprocessing pipeline of the NIRS data.
Data analysis	Further data analysis was performed in custom code in MATLAB using component written in C and Java for computational efficiency and interoperability. An executable software version of the presented method is deposited in the Github community repository https://github.com/jandreu/xMPVA and Zenodo: https://doi.org/10.5281/zenodo.4644294.

For manuscripts utilizing custom algorithms or software that are central to the research but not yet described in published literature, software must be made available to editors and reviewers. We strongly encourage code deposition in a community repository (e.g. GitHub). See the Nature Research guidelines for submitting code & software for further information.

Data

Policy information about availability of data

All manuscripts must include a data availability statement. This statement should provide the following information, where applicable:

- Accession codes, unique identifiers, or web links for publicly available datasets
- A list of figures that have associated raw data
- A description of any restrictions on data availability

Data from the analysis of this manuscript is publicly available at Princeton Data repository http://arks.princeton.edu/ark:/88435/dsp01xs55mf543. Data used to generate figures 4, 5, 6 of the main manuscript, and 1, 2, 3 of the supplementary materials are provided with the paper.

Field-specific reporting

Please select the one below that is the best fit for your research. If you are not sure, read the appropriate sections before making your selection.

☒ Life sciences ☐ Behavioural & social sciences ☐ Ecological, evolutionary & environmental sciences

For a reference copy of the document with all sections, see nature.com/documents/nr-reporting-summary-flat.pdf

Life sciences study design

All studies must disclose on these points even when the disclosure is negative.

Sample size	19 subjects were included in the final data-analysis after data exclusions.
Data exclusions	Twenty-five (25) infants were recruited for this study, 19 were included in the final data analyses, three infants excluded due to poor optical contact (e.g., due to a large amount of dark hair); these individual subject exclusions were made based on experimental notes or from observation of the recordings. The decision to include or exclude each subject was made once and before the data were analyzed to reduce the possibility of experimental bias and 3 for failing to watch the video to criterion.
Replication	The results from the finding of this paper are reproducible using the same data, implementing the method described in section IV, and following the same evaluation process suggested in this section. Data is already publicly available from our institutional repository, and executable software has been made available in a public repository that includes the analysis data of this manuscript as a demo, and it can be used to reproduce the same analysis. Source data from all figured representing data or results are included in the manuscript submission.
Randomization	Stimuli randomization:<br>A randomly ordered combination of auditory and/or visual stimuli were presented in unimodal signal trials and each separated by a jittered inter-stimulus interval (ISI) (1–1.5 s): two non-speech auditory stimuli (well-described as a honk, like from a clown horn, and an unusual rattle sound) and/or two visual stimuli that are a red cartoon smiley face that was presented in two different events or ways (entering a white box from either the top or the bottom, moving into the box to touch the opposite side of the box in 500 ms, and then exiting the box in the same side that it entered from in another 500 ms).<br><br>Control from influential factors or covariates:<br>The experiment was conducted in a darkened room with dark floor-to-ceiling curtains surrounding the infant and the caregiver. Only the monitor (Tobii eye tracker) was visible to the infant because all other equipments (e.g., speakers, computers) were on the other side of the curtains and out of sight. During the experiment, the infant sits on a caretaker's lap in a darkened room and is surrounded by a black curtain to reduce visual distraction and separate the participant from the experimenter. Caretakers were instructed to refrain from influencing their infant, only providing comfort if needed. Infants watched the video until they consistently stopped looking, became fussy or inattentive.
Blinding	Blinding is not a significant concern for this study as the participants are 6-month old babies. Regardless, to address any possible bias, all recruited babies were born no more than three weeks before their due date, had no major health problems or surgeries, no history of ear infections, nor known hearing or vision difficulties.

Reporting for specific materials, systems and methods

We require information from authors about some types of materials, experimental systems and methods used in many studies. Here, indicate whether each material, system or method listed is relevant to your study. If you are not sure if a list item applies to your research, read the appropriate section before selecting a response.

Materials & experimental systems

n/a	Involved in the study	
☒	☐	Antibodies
☒	☐	Eukaryotic cell lines
☒	☐	Palaeontology and archaeology
☒	☐	Animals and other organisms
☐	☒	Human research participants
☒	☐	Clinical data
☒	☐	Dual use research of concern

Methods

n/a	Involved in the study	
☒	☐	ChIP-seq
☒	☐	Flow cytometry
☒	☐	MRI-based neuroimaging

Human research participants

Policy information about studies involving human research participants

Population characteristics	Twenty-five (25) infants were recruited for this study (mean age = 5.7, SD = 0.61 months, ten female, 2/25 infants were identified as Hispanic and 23 infants were identified by their parents as Caucasian and two were identified as mixed race, Caucasian + Asian, Caucasian + Native American + Black). Of these infants, 19 were included in the final data analyses, with three infants excluded due to poor optical contact (e.g., due to a large amount of dark hair; these individual subject exclusions were made based on experimental notes or from observation of the recordings.
Recruitment	Infants were recruited through the database of interested participants from the Rochester Baby Lab and were born no more than three weeks before their due date, had no major health problems or surgeries, had no history of ear infections, or had known hearing or vision difficulties. The decision to include or exclude each subject was made once and before the data were analyzed in order to reduce the possibility for experimental bias) and 3 for failing to watch the video to criterion.
Ethics oversight	Written consent was obtained from a legal guardian for each infant before the experiment. The Institutional Review Board approved this consent procedure and the experimental methods of the University of Rochester (USA).

Note that full information on the approval of the study protocol must also be provided in the manuscript.
